# Comparative genomics reveals convergent signals associated with the high metabolism and longevity in birds and bats

**DOI:** 10.1098/rspb.2024.1068

**Published:** 2024-08-28

**Authors:** Yuki Matsuda, Takashi Makino

**Affiliations:** ^1^ United Graduate School of Agricultural Science, Tokyo University of Agriculture and Technology, Saiwai-cho, Fuchu-shi, Tokyo 183-8509, Japan; ^2^ Graduate School of Life Sciences, Tohoku University, Aoba-ku, Sendai 980-8578, Japan

**Keywords:** lifespan, longevity, flying species, convergent evolution

## Abstract

Birds and bats have long lifespans relative to their body size compared with non-flying animals. However, the genomic basis associated with longer lifespan of flying species despite their higher metabolism was unclear. In this study, we hypothesized that genes involved in the regulation of metabolism and lifespan changed with the acquisition of flight and searched for genes that show specific evolutionary patterns in flying species. As a result, we identified several genes that show different evolutionary rates in bird and bat lineages. Genes in pathways involved in lifespan regulation were conserved in birds, while they evolved at an accelerated rate in bats. We also searched for genes in which convergent amino acid substitutions occurred in birds and bats and found such substitutions in genes involved in cancer, reactive oxygen species control and immunity. Our study revealed genomic changes associated with the acquisition of flight in birds and bats and suggested that multiple genes involved in the regulation of lifespan and metabolism support both high metabolism and longevity in flying species.

## 1. Introduction

Lifespan is one of the fundamental life-history traits of organisms that shows remarkable diversity among species. Interspecific differences in lifespan are the result of adaptation to different environments or differences in life-history strategies related to growth and reproduction [1–3]. Therefore, lifespan correlates with other life-history traits. For example, vertebrate species with larger body size are generally known to have longer lifespan [4]. Similarly, species with lower metabolic rates tend to have extended lifespan [5]. However, many species have lifespan that are much longer or shorter than would be predicted from their body size and metabolic rate. Elucidating how such deviations occur will facilitate the understanding of the mechanisms that control lifespan and its evolution.

In flying species, such as birds and bats, many species have longer lifespans than expected, given their body size and metabolic rate [6,7]. For example, Brandt’s bats (*Myotis brandtii*) live up to 41 years [8], which is remarkable, compared with the maximum lifespan of 6 years for mice (*Mus musculus*), which are roughly the same size. The ability to fly is thought to have enabled these species to escape predation and unfavourable environments, thus they can evolve to reduce extrinsic mortality and increase their lifespan [7,9,10]. In environments with high extrinsic mortality, organisms evolve to have short lifespan because it is more adaptive to invest in early reproduction than to invest in long-term survival [11,12]. Moreover, when extrinsic mortality is low, organisms are thought to evolve to live longer by investing in long-term survival [13].

However, from a physiological perspective, the oxidative stress produced by the high metabolism required for flight may shorten lifespan. Reactive oxygen species (ROS) are generated as a product of oxygen metabolism in mitochondria [14,15]. ROS damage DNA, proteins and membrane lipids, thereby accelerating aging. Therefore, species with higher metabolic rates tend to have shorter lifespans [16–18]. Flight requires the highest metabolic rate [19,20] and has the highest oxygen demand of all vertebrate locomotion forms [21]. Although it is expected that the high metabolic rate of flight to shorten the lifespan of birds and bats [22], they actually live longer than other terrestrial animals. Therefore, birds and bats may have undergone common genetic changes that have allowed them to evolve to live longer, effectively suppressing the effects of ROS due to their high metabolism [1].

In recent years, comparative genomics has been used to explore the genetic factors associated with interspecific differences in lifespan. Genome sequences of long-lived bat lineage, including Brandt’s bats, suggested that specific amino acid substitutions in genes encoding growth hormone receptors (*GHR*, *IGF-1R*) are responsible for the evolution of body size and longevity in these species [23]. In bowhead whales (*Balaena mysticetus*) and naked mole rats (*Heterocephalus glaber*), it is suggested that the species-specific amino acid substitutions in *UCP1*, a gene involved in heat production, caused the evolution of longevity through metabolic regulation [24,25]. Pan-mammalian comparative genomics has revealed that multiple pathways are under purifying selection; this observation is based on the analysis of genes whose evolutionary rates correlate with lifespan [26]. These results suggest that genetic changes involved in the evolution of longevity stem from the preferential selection of mutations that favour long-term body maintenance. In particular, it is thought that common genetic changes have occurred in birds and bats, resulting in both high metabolism and long lifespan [1]; however, this hypothesis has not been tested.

In this study, we used amniotes species for which high-quality genomes have been sequenced to conduct a comprehensive search for genes that exhibit specific evolutionary patterns in birds and bats. The aim of this study is to identify genetic changes responsible for the evolution of high metabolism and long lifespan in birds and bats. Notably, we detected genes with accelerated or conserved evolutionary rates in bird or bat lineages, compared with other species, as well as genes in which convergent amino acid substitutions occurred in birds and bats. We found that multiple genes and pathways involved in the regulation of lifespan and metabolism show unique evolutionary patterns in birds and bats. This study revealed that genes and pathways involved in the regulation of lifespan and metabolism are targeted for selection with the acquisition of flight, suggesting that these genetic changes support both longevity and high metabolism in flying species.

## 2. Material and methods

### (a) Acquisition of genomic data and inference of orthologues

All genomic data were obtained from National Center for Biotechnology Information (NCBI, final access on 8 June 2023). Protein sequences, coding sequence (CDS) and GTF files were obtained for all Amniota species. The sequences were assembled at the chromosomal level and protein-coding regions were identified. We obtained genomic data for crocodilians, the closest extant lineage to birds, bats and a flying species. The sequences were assembled at the scaffold level for further analysis. In total, genomic data were obtained for 132 species, including 15 orders of mammals, 10 orders of birds and three orders of reptiles (electronic supplementary material, data S1). We confirmed that this subsampling of species is representative of amniote species in terms of longevity traits (electronic supplementary material, text S1, figure S1). For each species, the longest protein sequence was selected as the representative sequence for each gene. For the CDS, those corresponding to the selected protein sequences were used. Using the selected protein sequences, homology searches using BLAST + 2.11.0 [27] were performed with the parameters ‘-evalue 1e − 4, -max_target_seqs 1’. Human sequences were used as reference sequences, and when a sequence had a reciprocal best hit with a human gene for each species, that gene was used as an orthologue.

### (b) Alignment and gene tree construction

MAFFT 7.487 [28] was used to align the amino acid sequences of the orthologues. Nucleotide sequence alignments were also generated from the amino acid sequence alignments and the nucleotide sequence of the CDS using in-house Python scripts. Residues that did not match the amino acids in the obtained sequence and those translated from the CDS, as well as in-frame stop codons in the CDS, were converted to ‘N’ representing unknown bases. All letters representing ambiguous amino acid residues were replaced with ‘X’ to signify unknown amino acid residues. This substitution was made because these residues are not supported by RERconverge, the tool used in subsequent analysis. The amino acid and nucleotide sequence alignments were trimmed in two steps using trimAl v. 1.4.1 [29]. In the first step, sequences that contained over 10% of the total sequence length in gaps were removed. In the second step, all loci containing gaps were removed. Genes for which at least three species were retained after trimming were used in subsequent analyses. Short sequences with a sequence length of less than 30 amino acids after trimming were excluded from the analysis because extremely short sequences may lead to false positives caused by stochastic fluctuations. Phylogenetic trees were estimated using IQ-TREE v. 2.2.0 [30] for each gene, and ModelFinder [31] was used for model selections.

### (c) Detection of genes with different evolutionary rates in bird and bat lineages

The R package RERconverge [32] was used to search for genes with altered relative evolutionary rates in each of the bird and bat lineages. RERconverge computed the relative evolutionary rate for each branch of the gene tree by comparing the amino acid substitution rate of the tree with the average of all genes. Using the “estimatePhangornTreeAll()” function implemented in RERconverge, a maximum-likelihood gene tree was constructed under the topology of the species tree, where the branch length represents the evolutionary rate determined by the number of amino acid substitutions. The topology of the species phylogenetic tree was obtained from TimeTree (http://timetree.org/, final access on 8 June 2023) [33]. The “getAllResiduals()” function was used to estimate the relative evolutionary rate in each branch by correcting the branch length of each gene with the average branch length of all genes.

Using the estimated relative evolutionary rates, we searched for genes whose relative evolutionary rates significantly differed between flying species and other species. The ‘correlateWithBinaryPhenotype()’ function was used to calculate the correlation statistics using genes for which at least 10 species were retained by ‘min.sp = 10, min.pos = 10’ option. We confirmed the small number of species in genes does not affect the statistical power of analysis (electronic supplementary material, figure S2). After corrections for multiple testing using the Benjamini–Hochberg (BH) method [34], those with corrected *p*-values below 0.05 were considered accelerated or conserved genes. Whether a gene is accelerated or conserved was determined by Rho, which represents the correlation coefficient of evolutionary rate shift. The significance of acceleration/conservation was determined by the rank of the adjusted *p*-values.

To validate evolutionary rate shifts in codon-based sequences, we used the branch model of codeml implemented in PAML [35] to search for genes with different $\omega =d_{\text{N}}/d_{\text{S}}$ , which represents the ratio of synonymous to non-synonymous substitution rates, in each bird and bat lineages. Using the sequence alignment of each gene and the gene tree estimated by IQ-TREE, we estimated $d_{\text{N}}/d_{\text{S}}$ by codeml using PAML. We calculated the log-likelihood of the M0 model [36], which assumes that all branches of the gene tree have the same $d_{\text{N}}/d_{\text{S}}$ , and the free model [35], which assumes that each bird and bat lineages have different $d_{\text{N}}/d_{\text{S}}$ from non-flying species. Among the genes for which the free model was accepted by the likelihood ratio test, we corrected for multiple testing using the BH method and selected genes with corrected *p*-values below 0.05 as those with significantly different evolutionary rates.

### (d) Enrichment analysis

Gene Set Enrichment Analysis of Kyoto Encyclopedia of Genes and Genomes (KEGG [37]) pathways was performed using clusterProfiler [38]. Against the background of all the genes used in the analysis, we used a rank-based approach to determine whether accelerated and conserved genes detected by RERconverge were enriched in specific functions or pathways. We regarded pathways with corrected *p*-values below 0.05 after the correction for multiple testing as significantly enriched.

### (e) Detection of convergent amino acid substitutions in birds and bats

To investigate the molecular convergence that occurred in each common ancestor of birds and bats, we used CSUBST [39] to detect convergent amino acid substitutions. CSUBST is a recent method for examining adaptive convergent amino acid substitutions that may have undergone positive selection. This is achieved by expanding the $d_{\text{N}}/d_{\text{S}}$ ratio to consider multiple combinations of branches in a phylogenetic tree and calculating $\omega_{c}$ , which represents the ratio of non-synonymous convergent substitution rates to synonymous convergent substitution rates. We ran CSUBST with the parameters ‘–exhaustive_until 1 –fg_exclude_wg yes –fg_stem_only yes’ using the codon alignment of each gene. Birds and bats were selected as foreground species. Based on previous studies [39], we selected genes that showed high convergent amino acid substitution rates with $\omega_{c}\geq 3$ . ${O_{C}}^{N}\geq 3$ was used as the threshold because $\omega_{c}$ may be overcalculated when the synonymous convergent substitution rate is low owing to the short branches in the gene tree even if the non-synonymous convergent amino acid substitution rate is low.

## 3. Results

### (a) Convergent evolutionary rate shift occurred in multiple pathways in flying species

**Figure 1.**
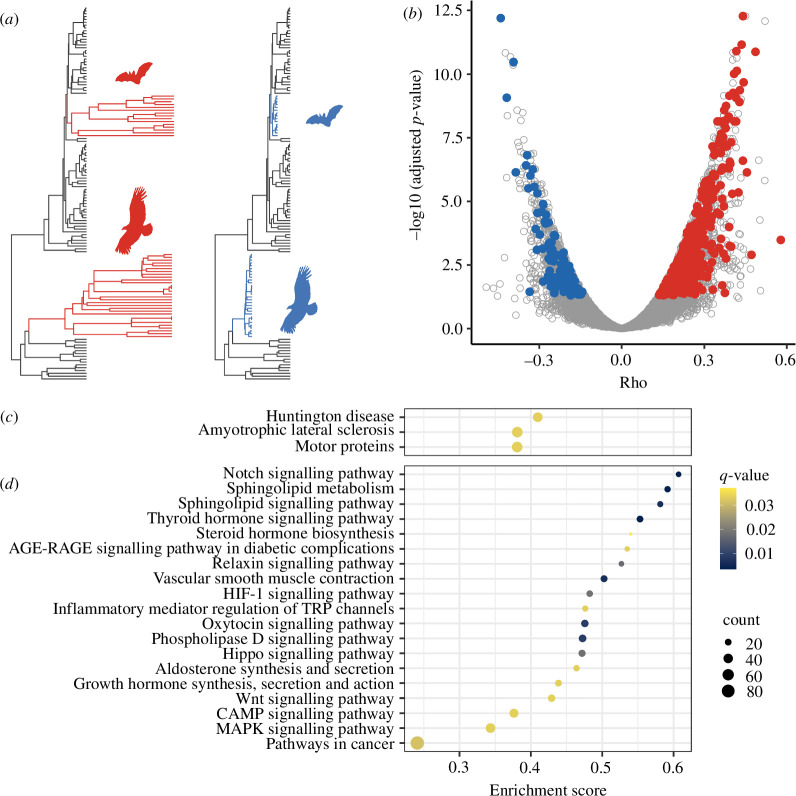
Evolutionary rate shift in birds and bats**.** (*a*) Schematic phylogenetic tree of accelerated (left) and conserved (right) genes in flying species. (*b*) Volcano plot of evolutionary rate shifts in flying species. The horizontal axis shows the Rho of evolutionary rate, with rightward indicating accelerated in flying species and leftward indicating conservative in flying species. Genes with adjusted *p*-values <0.05 after multiple correction for both RERconverge and PAML are indicated by red and blue circles. (*c,d*) Pathway enrichment analysis of accelerated genes (*c*) and conserved genes (*d*). The horizontal axis represents the degree of gene enrichment taking values ranging from 0 to 1 [40].

To explore the evolutionary rate shift associated with the acquisition of flight, we used RERconverge to detect genes that have different relative evolutionary rates between non-flying species and flying species (figure 1*a*, electronic supplementary material, data S2). We performed homologous gene searches against human genes in 132 amniotes lineages, including 30 bird and 15 bat species, and analysed 18 852 protein-coding genes. After correction for multiple testing using BH’s method, 2042 genes were found to be accelerated in flying species and 1337 genes were found to be conserved. The most strongly associated accelerated gene was *RPGRIP1L* ( Rho=0.4500812 ; adjusted *p*-value $<5.34\times 10^{-13}$ ). We also detected *UCP1*, *SPAG16*, *CCDC42* and *ITPR1* as accelerated genes (electronic supplementary material, data S2). *UCP1*, which regulates heat production and metabolism, is known to be associated with the evolution of lifespan [24,25]. *SPAG16*, *CCDC42* and *ITPR1* are genes whose evolutionary rates positively correlate with the longevity quotient in mammals [41,42]. This indicates that our analysis selecting flying species as the foreground captures a part of genes associated with lifespan. The most strongly associated conserved gene was *ANKRD11* ( Rho=-0.4404185 ; adjusted *p*-value $<6.41\times 10^{-13}$ ). *ANKRD11* acts as a coactivator of p53 which has a function as a tumour suppressor [43].

To further investigate the selection of protein-coding genes in flying species, we additionally applied an analysis with codeml implemented in PAML, which compares non-synonymous substitution rates ( $d_{\text{N}}$ ) and synonymous substitution rates ( $d_{\text{S}}$ ) in codon sequences. We identified genes with significantly greater or smaller $d_{\text{N}}/d_{\text{S}}$ in bird and bat branches compared with branches of non-flying species. After correction for multiple testing, 838 genes were found to be accelerated in flying species and 397 genes were found to be conserved (electronic supplementary material, data S3). We examined overlap with genes detected by RERconverge and detected 397 accelerated genes and 116 conserved genes (figure 1*b*, electronic supplementary material, figure S3*a*,*c*). The overlaps between genes detected by RERconverge and PAML were significantly more than expected by chance (permutation *p*‐value < 0.001, electronic supplementary material, figure S3*b*,*d*). *ANKRD11* remained the most strongly conserved gene. *PKD1* was detected as the next most conserved gene in the flying species. *PKD1* encodes a membrane protein, polycysteine, which regulates metabolism in mitochondria through the regulation of intracellular calcium homeostasis [44].

To examine functional trends of accelerated and conserved genes in flying species, enrichment analysis of KEGG pathways was performed using clusterProfiler with a rank-based method of *p*-values calculated with RERconverge. Genes with accelerated evolutionary rates in flying species were associated with Huntington’s disease (HD), amyotrophic lateral sclerosis (ALS) and motor proteins (figure 1*c*, electronic supplementary material, data S4). Genes with conserved evolutionary rates in flying species were associated with multiple signalling pathways, including pathways in cancer, as well as vascular smooth muscle contraction, inflammatory mediator regulation of TRP channels, growth hormone synthesis, secretion and action (figure 1*d*, electronic supplementary material, data S4). Considering that genes involved in the pathways in cancer were conserved in flying species, we compared the evolutionary rates between known cancer genes from OncoKB (https://www.oncokb.org/). We found that oncogenes were significantly conserved in flying species while tumour suppressor genes had no significant difference compared to all genes (Wilcoxon rank-sum test, electronic supplementary material, figure S4). This result shows that the enrichment of cancer-related pathways in conserved genes of flying species is due to the conservation of the oncogenes, not the tumour suppressor genes.

### (b) Divergent evolutionary rate shift of genes involved in the longevity regulating pathway

**Figure 2.**
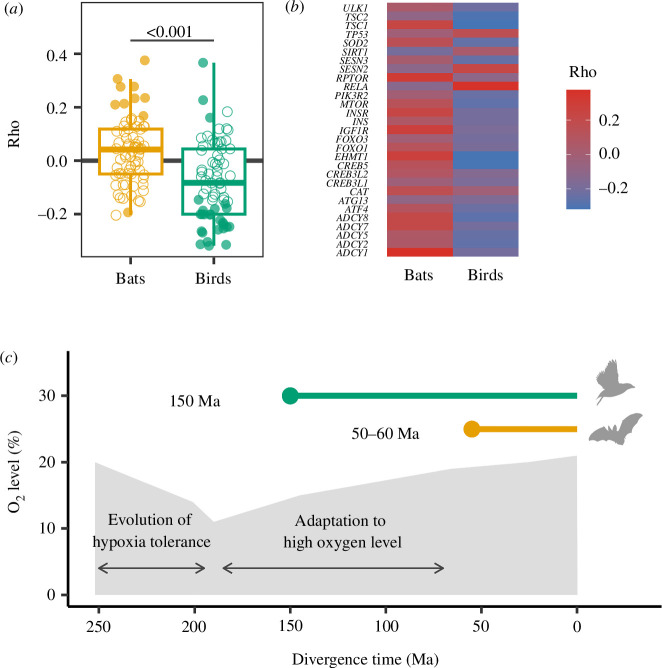
Evolutionary rate shift of genes in the longevity regulating pathway in birds and bats. (*a*) Boxplot of Rho distribution showing the difference between birds and bats. Orange and green circles represent genes with significant and white circles with non-significant *p*-values. (*b*) Heat map showing Rho of genes in the longevity regulating pathway for which significant acceleration or conservation was detected using RERconverge in birds or bats. Grey panels in ‘Birds’ and ‘Bats’ show that the genes were not significantly accelerated/conserved. (*c*) Increase and decrease in atmosphere oxygen level and divergence time of birds and bats. The horizontal axis represents the divergence time (Ma). The vertical axis and grey areas in the figure represent the estimated atmospheric oxygen level [45]. The green and orange lines show the divergence of birds and bats, respectively.

To examine evolutionary rate shifts in flying species for genes known to be involved in lifespan regulation, we performed RERconverge with birds and bats as foregrounds, respectively (electronic supplementary material, data S5, data S6). We examined Rho, which represents the correlation coefficient of acceleration/conservation, for 89 genes in the longevity regulating pathway of KEGG (hsa04211). These genes were conservative in the bird lineage while accelerated in the bat lineage ( $p-value=4.27\times 10^{-4}$ , Wilcoxon signed rank test, figure 2*a*). This pattern was not observed across all genes (electronic supplementary material, figure S5a), highlighting the divergent evolutionary rate shift in this pathway. Of the 89 genes, a significant evolutionary rate shift (adjusted *p*‐value < 0.05) was detected for 29 genes in either the bird or bat lineages. Among these genes, a large portion (20/29) exhibit acceleration in bat lineage, whereas the majority (25/29) were conserved in bird lineage (figure 2*b*).

We additionally examined the evolutionary rate shift pattern in the longevity regulating pathway—multiple species in KEGG (hsa04213) and known ageing-related genes of human in GenAge (https://genomics.senescence.info/genes/index.html). We observed the consistent pattern in the longevity regulating pathway—multiple species (electronic supplementary material, figure S5b), indicating that evolutionary rate shifts are divergent in genes involved in longevity in amniotes. However, it did not apply to GenAge genes (electronic supplementary material, figure S5c). GenAge genes are involved in multiple pathways, hence this result suggests that certain pathways correspond to the evolution of lifespan in flying species.

### (c) Convergent amino acid substitutions in birds and bats

Phenotypic convergence is caused by molecular changes, which often coincide with the molecular convergence of gene sequences [45,46]. We searched for genes with convergent amino acid substitutions in each common ancestor of birds and bats. Using the same thresholds applied in the previous study ( $\omega_{c}\geq 3,{O_{C}}^{N}\geq 3$ ) [39], we identified seven genes (*SLC34A2*, *MYO5C*, *LIPG*, *SEC14L2*, *ADGRF5*, *RYR2* and *SAMD9L*) with accelerated rates of convergent amino acid substitutions (table 1).

**Table 1.** List of genes for which convergent amino acid substitutions were detected.

gene	description	$\omega_{C}$	${O_{C}}^{N}$
*SLC34A2*	solute carrier family 34 member 2	159.5139	3.2956
*MYO5C*	myosin VC	27.0059	4.0729
*LIPG*	lipase G, endothelial type	9.4146	3.9264
*SEC14L2*	SEC14 like lipid binding 2	6.3554	3.0470
*ADGRF5*	adhesion G protein-coupled receptor F5	6.1944	3.4874
*RYR2*	ryanodine receptor 2	5.1038	3.1175
*SAMD9L*	sterile alpha motif domain containing 9 like	4.7899	4.1500

**Figure 3.**
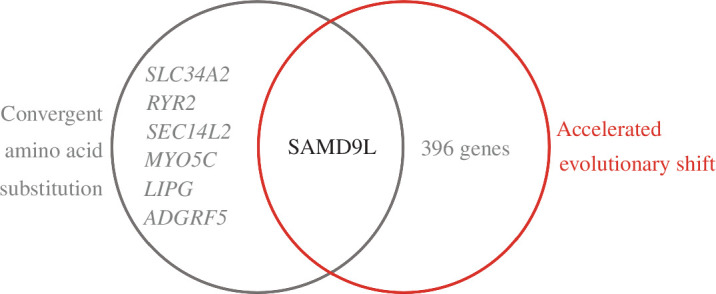
Venn diagram showing the overlap between genes detected by CSUBST and evolutionary shift analysis.

To determine whether genes that underwent convergent amino acid substitutions with the acquisition of flight caused evolutionary rate shifts in bird and bat lineages, we examined the intersection of genes detected by CSUBST with those detected by RERconverge and PAML. We found that among the seven genes detected by CSUBST, *SAMD9L* evolved at an accelerated rate in the lineage of flying species (figure 3).

## 4. Discussion

In this study, we identified genes in each of the bird and bat lineages that have different evolutionary rates, compared with those of other amniote species. The evolutionary rate of these genes may have altered as a result of changes in selection pressure associated with the independent acquisition of flight. Based on the hypothesis that common genetic changes contributing to both high metabolism and long lifespan occurred with the acquisition of flight, we searched for genes involved in the regulation of metabolism and lifespan.

### (a) Multiple pathways are under purifying selection in flying species

We explored genes which show convergent evolutionary rate shift in birds and bats, and detected multiple genes that are accelerated or conserved in flying species (figure 1*b*). Pathway enrichment analysis revealed that conserved genes in flying species are enriched in some signalling pathways including pathways in cancer (figure 1*d*). This result suggests that these pathways are under purifying selection in flying species. A previous study has reported that genes involved in the control of cancer evolve slower in long-lived mammals [26]. In our study, we treated mammals as background species, so cancer-related pathways in birds and bats are thought to be undergoing stronger purifying selection than long-lived mammals. One explanation is that as birds and bats live longer and lay or give birth at older ages, the function of cancer-suppressing genes has become more important. Alternatively, it can be viewed as the result of adaptation to hypoxia or high ROS. The environment around cancer cells is known to have lower oxygen and higher ROS levels than normal cells [47], and genes that evolved in birds and bats by adapting to hypoxia and high ROS may also be involved in cancer cells’ survival in the tumour environment.

Some genes showed a strong signal of convergent evolutionary rate shift in birds and bats. *ANKRD11* was detected as the most conserved gene. This gene encodes an ankryin repeat domain-containing protein which acts as a coactivator of a tumour suppressor [43]. Purifying selection of this gene may also reflect the importance of tumour suppression in flying species. *PKD1* was also detected as another highly conserved gene in the flying lineages. *PKD1* is involved in protection against oxidative stress in a variety of cells, including neurons [48], intestinal cells [49] and osteoblasts [50]. The expression level of *PKD1* correlates with cell viability under oxidative stress [50]. *PKD1* has been detected as a conserved gene in both birds and bats, and sequence mutations may be suppressed owing to functional constraints for cell protection under flight-induced oxidative stress.

### (b) Motility-related pathways are accelerated in flying species

We found that genes with accelerated evolutionary rates in flying species were enriched in motility-related pathways, such as motor protein (figure 1*c*). HD is a neurodegenerative disorder with a distinct phenotype, which includes incoordination and behavioural disturbances [51]. ALS is a neurodegenerative disease of the motor system [52]. Both HD and ALS are diseases related to motility, suggesting that motility-related genes are the subject of accelerated evolutionary rate shifts in flying species. Flight is a form of locomotion with high energy consumption, and this energy expensiveness provides a common selection pressure for efficient locomotion [53]. The functional enrichment of accelerated genes in the motility-related pathways may be caused by the positive selection of genes associated with physical activity to optimize the locomotion of flying species.

A previous study reported that genes whose evolutionary rates correlate with lifespan and other life-history traits in primates are enriched in aging-related pathways. These genes are also enriched in motility-related pathways, such as cardiac muscle contraction. It is possible that genes involved in motor function are pleiotropically involved in lifespan.

### (c) The evolutionary response of the longevity regulating pathway is divergent in flying species

The evolution of lifespan-related genes is interesting given that flying species have a longer life span than other species of the same body size. We investigated the evolutionary rate shift of genes in the longevity regulating pathway in birds and bats. We found that the relative evolutionary rates of genes involved in this pathway are conserved in birds, while they are accelerated in bats (figure 2*a*,*b*). This is interesting because it indicates that birds and bats have different evolutionary responses to the evolution of longevity relative to body size. Genomic analysis of Brandt’s bats (*M. brandtii*), known as long-lived bats, reports lineage-specific amino acid substitutions in genes involved in the insulin signalling pathway that act as the starting point for the longevity regulating pathway [23,54]. Our analysis also detected the accelerated evolutionary rate shift of genes in this pathway such as *IGF1R* and *INSR* in bats (figure 2*b*). This result supports the previous studies that suggest the importance of the insulin pathway in longevity of bats. Although more detailed studies are needed in the future, the evolutionary trends of sequences in lifespan-related genes may not necessarily be common among long-lived species.

Given the more recent acquisition of flight in bats, compared with birds (about 150 Ma for birds [55,56] and 50–60 Ma for bats [57,58]), it is possible that genes underwent adaptive changes with flight acquisition and later became conserved due to functional constraints, thereby maintaining high metabolism and longevity in the long-term (figure 2*c*). One of the factors that facilitated the evolution of tolerance of oxidative damage in birds was a significant increase or decrease in atmospheric oxygen level from the P–T boundary (about 250 Ma) to the K–T boundary (about 66 Ma [59,60]). The generation of ROS due to hypoxic stress associated with the decrease in atmospheric oxygen level [61] during this period and the subsequent oxidative stress caused by the increase in oxygen level may have resulted in strong selective pressure on the oxidative stress response of the organisms. The divergence of birds coincides with this period of increase and decrease in oxygen level [55]. It is possible that the system of oxidative stress response acquired during this period mitigates the oxidative stress associated with flight [62]. While birds maintained their oxidative stress response system through purifying selection, non-flying animals may have reduced the selection pressure on their oxidative stress response system once the atmospheric oxygen level stabilized. The divergence in bats occurred after the atmospheric oxygen level stabilized [57], and the need to control oxidative stress associated with the acquisition of flight may have again exerted selection pressure on the pathways involved in the oxidative stress response, resulting in accelerated evolution.

### (d) Molecular convergence in flying species is associated with immunity and the regulation of oxidative stress

We used CSUBST to search for accelerated convergent amino acid substitutions in birds and bats and identified seven genes. Among these genes, *SAMD9L* was also detected as an accelerated gene in flying species. This gene encodes a cytoplasmic protein that acts as a tumour suppressor but also plays a key role in cell proliferation and the innate immune response to viral infection (provided by RefSeq [63]). Bats and birds are the natural hosts of many zoonotic pathogens [64], thus resistance to viruses may be important for the longevity of birds and bats. It is possible that birds and bats have enhanced resistance to pathogens through convergent amino acid substitutions associated with the acquisition of flight, and that accelerated evolution of this gene reflects the evolution of resistance to viruses. However, we found no other genes in which these two convergent signals were detected. It may not be general that the convergent amino acid substitutions cause evolutionary shifts of genes.

Two of these genes, *SLC34A2* and *LIPG*, are involved in cancer in humans and in hypoxic responses and ROS regulation in cancer cells [47,65]. Cancer cells exhibit higher metabolic rates and higher ROS levels as a result of hypoxia, compared with normal cells [66]. Moreover, it is possible that genes involved in the regulation of high metabolism and ROS in cancer cells are associated with the response to the oxidative stress in flying species. *SLC34A2* is a gene encoding a sodium-dependent phosphate transporter. It is involved in the cell cycle and is highly expressed in colon cancer cells [47]. SLC34A2 works cooperatively with ROS and HIF-1 to activate downstream hypoxia response pathways [65]. *LIPG* is a gene encoding a phospholipase, which has been implicated in cancer development and progression [67]. Oxidative stress upregulates *LIPG* expression and protects cells from oxidative stress by accumulating lipid droplets in tumour cells [68]. Previous studies have shown that cancer suppression is important, particularly in species with large body size and longevity [25,26,69]. It is unclear how important cancer suppression is in species with long lifespan relative to body size, such as birds and bats, compared with species with large body sizes. Although recent studies have suggested that cancer suppression is also involved in longevity in birds and bats [70,71], the convergent amino acid substitutions detected in this study may be associated with the selection for the control of flight-associated oxidative stress, rather than selection in favour of cancer suppression.

*RYR2* is a gene encoding a ryanodine receptor in the cardiac sarcoplasmic reticulum and may be involved in the regulation of ROS in cardiomyocytes. It regulates the release of calcium ions from the sarcoplasmic reticulum of cardiomyocytes. The released calcium ions translocate to the mitochondria and promote ATP production for myocardial contraction [72]. Increased *RYR2* activity promotes the generation of ROS associated with oxygen metabolism in the mitochondria. Moreover, the increased ROS oxidizes RYR2, further increasing its activity and compromising intracellular calcium ion homeostasis [73]. Therefore, proper regulation of RYR2 activity is important for ROS suppression, and the convergent amino acid substitutions that occurred in *RYR2* in birds and bats may have contributed to the regulation of RYR2 activity and ROS.

### (e) Limitations of the study

In this study, we identified accelerated and conserved genes in each lineage of birds and bats. We also identified genes that underwent accelerated amino acid convergence with the acquisition of flight in birds and bats. While these genes may be involved in longevity and high metabolism in flying species, the physiological consequences of each genetic change are unknown. We found that some known longevity-related genes show evolutionary rate shifts in flying species, however, our analysis did not necessarily disentangle adaptations associated with longevity from those linked to flight. This does not deny that these evolutionary rate shifts come from the adaptations related to the other traits. Although it is difficult to gain insight into the evolution of gene function and molecular mechanisms from informatics analysis of genomic data alone, further studies combining different methods can provide more detailed insights into the evolutionary basis and mechanisms. For example, a comparison of gene expression levels may be useful for assessing the mechanism of action and evolution of a gene. With future accumulation of gene expression data, it is expected that comparisons of expression levels among species will further elucidate the molecular mechanisms involved in longevity and high metabolism in flying species.

## Data Availability

Codes for this research are available via Zenodo [74]. Supplementary material is available online [75].
